# Association of HLA Class-I and Inhibitory KIR Genotypes in Gabonese Patients Infected by Chikungunya or Dengue Type-2 Viruses

**DOI:** 10.1371/journal.pone.0108798

**Published:** 2014-09-29

**Authors:** Caroline Petitdemange, Nadia Wauquier, Jean-Michel Jacquet, Ioannis Theodorou, Eric Leroy, Vincent Vieillard

**Affiliations:** 1 Sorbonne Universités, UPMC, Univ Paris 06, CR7, CIMI-Paris, Paris, France; 2 INSERM, U1135, CIMI-Paris, Paris, France; 3 Centre International de Recherches Médicales de Franceville, Unité des maladies Emergentes, Franceville, Gabon; 4 Metabiota Inc., San Francisco, California, United States of America; 5 AP-HP Hôpital Pitié-Salpêtrière, Département d'Immunologie, Paris, France; 6 IRD, Maladies Infectieuses et Vecteurs: Ecologie, Génétique, Evolution et Contrôle, Montpellier, France; 7 CNRS, ERL8255, CIMI-Paris, Paris, France; Karolinska Institutet, Sweden

## Abstract

**Background:**

Natural killer (NK) cells provide defense in the early stages of the immune response against viral infections. Killer cell immunoglobulin-like receptors (KIR) expressed on the surface of NK cells play an important role in regulating NK cell response through recognition of human leukocyte antigen (HLA) class I molecules on target cells. Previous studies have shown that specific KIR/ligand combinations are associated with the outcome of several viral infectious diseases.

**Methods:**

We investigated the impact of inhibitory and activating KIR and their HLA-class I ligand genotype on the susceptibility to Chikungunya virus (CHIKV) and Dengue virus (DENV2) infections. From April to July 2010 in Gabon, a large outbreak of CHIKV and DENV2 concomitantly occurred in two provinces of Gabon (Ogooué-Lolo and Haut-Ogooué). We performed the genotypic analysis of KIR in the combination with their cognate HLA-class I ligands in 73 CHIKV and 55 DENV2 adult cases, compared with 54 healthy individuals.

**Results:**

We found in CHIV-infected patients that KIR2DL1 and KIR2DS5 are significantly increased and decreased respectively, as compared to DENV2^+^ patients and healthy donors. The combination of KIR2DL1 and its cognate HLA-C2 ligand was significantly associated with the susceptibility to CHIKV infection. In contrast, no other inhibitory KIR-HLA pairs showed an association with the two mosquito-borne arboviruses.

**Conclusion:**

These observations are strongly suggestive that the NK cell repertoire shaped by the KIR2DL1:HLA-C2 interaction facilitate specific infection by CHIKV.

## Introduction

Chikungunya virus (CHIKV) and Dengue virus (DENV) are two mosquito-borne arboviruses transmitted by the *Aedes* genus. The diseases caused by these viruses have much in common in terms of symptoms, incubation period, clinical course, and symptomatic treatments. Both viruses have been recently proven responsible for major outbreaks leading to serious health and economical problems, and the rapid geographical expansion of their vector could potentially lead to a worldwide increased risk within non-immune populations [1], [2]. However, unlike CHIKV, DENV strains are divided into four different serotypes (DENV1 to DENV4), which only confer short-term partial cross-protection against other strains, and contribute to the development of severe forms of Dengue fever (Dengue haemorrhagic fever/Dengue shock syndrome) [3]. Through active surveillance of acute febrile syndrome in Gabon, CHIKV and serotype 2 DENV (DENV2) were detected between 2007 and 2010, and have caused together a large simultaneous outbreak centered on Franceville in southeast Gabon in 2010 [4].

Although natural killer (NK) cells hold a central role early after number of viral infections, not only for viral containment but also for timely and efficient induction of adaptive responses, their role in the control of CHIKV and DENV2 infections is still poorly documented [5], [6]. NK cells are controlled by a combination of activating and inhibitory receptors, and the integration of signals induced upon ligation of these receptors determines whether they become activated. These receptors include the killer cell immunoglobulin-like receptors (KIR) that encode for a family of highly polymorphic genes, and individual KIR haplotypes differ in number and identity of genes [7]. Expression of KIR receptors is very complex and controlled by a stochastic mechanism that shuts off expression of some receptors and not others in individual cells thereby allowing different NK cell clones to recognize different targets [8]. It is therefore unlikely for two unrelated individuals to share the same KIR genes or haplotypes and express the receptors. The KIR receptors are type I integral membrane glycoproteins that are usually expressed on the cell surface as monomers. The KIR receptors are named according to the number (i.e. 2 or 3) of Ig-like domains present in the extracellular region as well as the length (i.e. L: long or S: short) of their cytoplasmic tails. Functionally, KIR-L carry one or two tyrosine-based inhibition motifs (ITIMs), which contribute to inhibitory signaling whereas, KIR-S have a lysine residue in their trans-membrane domain that is required for pairing with the tyrosine-based activation motif (ITAM) - containing adaptor DAP12 [9]. The KIR family now includes seven inhibitory KIRs and six activating KIRs, in addition to KIR2DL4, which is an unusual activating member of the KIR family with inhibitory potential. KIRs bind polymorphic major histocompatibility complex (MHC) class-I molecules. For instance KIR2DL1/KIR2DS1 and KIR2DL2/KIR2DL3/KIR2DS2 bind group 2 (C2) and group 1 (C1) HLA-C alleles, respectively, whereas, KIR3DL1 recognizes HLA-Bw4 epitopes [8]–[10]. Besides their role in inhibiting NK cell function, combinations of KIR and HLA molecules also play an essential role during NK education, to establish self-tolerance and to shape the KIR repertoire of fully functional NK cells. Indeed functional maturation of NK cells requires specific interaction with MHC class I molecules [11]. However, MHC class I genes map to chromosome 6 whereas KIR genes map to chromosome 19 [7]. Therefore, the inheritance of each group of genes and the expression of the receptors and their ligands are physically independent of one another. It has become increasingly clear that the strength of KIR-HLA interactions has functional significance, and can influence the susceptibility to or the outcome of various infectious diseases, as previously shown for human immunodeficiency virus type 1 (HIV-1) and hepatitis C virus (HCV) [12], [13], yet no such associations have been uncovered in the context of CHIKV and DENV infections. Therefore, this study was undertaken to determine the impact of inhibitory KIR and their HLA-class I ligand genotype on the susceptibility to CHIKV and DENV2 infections.

## Materials and Methods

### Ethics statement

The research we report here was conducted in accordance with the principals expressed in the Declaration of Helsinki and was approved by the relevant French and Gabonese institutional ethics panels. Following the 2010 Gabon outbreak, the public-health response was based on cooperation between the Gabonese Ministry of Health (MoH) and “Centre International de Recherches Médicales de Franceville” (CIRMF). All eligible participants were aged ≥18 years provided informed consent, but given the urgency of diagnosis and according to the MoH directives; only individual oral consent was required for sampling. All results were confidentially transmitted to the MoH. This procedure was approved by an Institutional Review Board (“Conseil Scientifique du CIRMF”) and the Regional Health Director (Authorization N°139, May 27, 2010). The institutional review board of the Pitié-Salpêtrière Hospital (Comité de Protection des personnes, Ile-de-France, Paris, France) also approved this study.

### Study population

Peripheral blood samples from 73 CHIKV-infected (male: 44.7%; age mean: 37.5 yr), and 55 DENV-2-infected (male: 25.7%; age mean: 34.6 yr) patients were obtained between April and July 2010 during the simultaneous outbreak of CHIKV and DENV-2, which occurred in two provinces (Ogooue Lolo and Haut Ogooue) of southeast Gabon, occupied predominantly by the rural rainforest Kaningi population. Peripheral blood samples were collected on suspected adult cases during the first five days after the following symptoms: fever (>38.5°C), arthralgias, myalgias, headaches, rash, fatigue, nausea, vomiting, diarrhea or bleeding. Patients who met the case definition were sampled and tested for various arboviral RNA genomes, as described [4]. Each sample was negative for Yellow, West Nile and Rift Valley fevers, or malaria. As controls, 54 Gabonese healthy individuals were selected to match for origin, age (mean: 36.0 yr), and sex (male: 41%) [14]. This healthy control group was sampled between 2001 and 2007, before the first outbreak of CHIKV and DENV2, which occurred in Gabon from March 2007 [4], [15].

### Viral RNA extraction and quantification

Diagnosis for CHIKV or DENV fevers was confirmed and quantification performed using a standard quantitative real-time reverse-transcription polymerase chain reaction (qRT-PCR) method. Briefly, RNA was extracted from 140 µL of plasma using the QIAamp Viral RNA Mini kit (Qiagen). cDNA was synthesized using qRT-PCR with a 9500 thermocycler (Applied biosystems), and mixing 25 µL of extracted RNA with 25 µL of High Capacity cDNA kit (Applied Biosystems). Five µL of newly synthesized cDNA was used as template in 25 µL of Taqman universal PCR Master Mix with specific CHIKV or DENV primers for a partial envelope (E1) gene sequence of CHIKV (692 bp, position 10 138–10829 nt) and a partial envelope (E) gene sequence of DENV-2 (758 bp, position 1503–2260 nt). Amplifications were run in duplicate in a 7500 Real-time PCR system (Applied Biosystem), as described [4], [5].

### KIR and HLA genotyping

DNA was extracted from whole blood using QIAamp DNA blood mini kit (Qiagen). Inhibitory KIR genotyping was performed by PCR using the KIR typing kit (Miltenyi Biotec, Inc) following manufacturer's instructions, as previously performed [14], and then confirmed by PCR using standard primers, and internal controls, as previously described [16]. HLA-Class I alleles were hybridized using LABType SSO kit (One Lambda). HLA sequences were read with a LABScan 200 (Luminex Technology) and computer-assisted HLA Fusion software.

### Statistical analysis

Fisher's exact test was used to compare results between healthy control and CHIKV^+^ or DENV^+^ patients. The P values of statistically significant differences were then corrected, by the formula P^n^ = 1−(1−P)^n^, where n is the number of comparisons [17].

## Results and Discussion

We studied variations in KIR polymporphic CMH class I genotypes and associations in CHIKV-, DENV2-infected patients and healthy individuals from Gabon.

Initial analysis of the KIR locus among all participants identified 37 different genotypes [18], [19]. Table 1 shows that the number of KIR genotypes in the different groups of patients varied from 18 to 21, with only KIR2DL4, KIR2DS4, KIR3DL3, KIR2DP1 and KIR3DP1 being detectable in all genotypes. All healthy and infected individuals possessed the framework genes (KIR2DL4, KIR3DL2 or KIR3DL3) [20]. This finding is biologically relevant to the studies showing that the NK cells lacking inhibitory receptors for self-MHC class I molecules could be hyporesponsive [21]. The frequency of individuals presenting an A/A genotype, containing only one activating gene (KIR2DS4), was higher in healthy individuals (24.0%) and DENV-2^+^ samples (16.7%), compared to CHIKV^+^ carriers (6.7%) (Table 1). The most represented AB/BB genotype in healthy donors (20.3%), genotype #15, was totally absent in CHIKV^+^ patients (p = 0.006), and only present in 1/31 (3.2%) DENV-2^+^ patient. Notably, in CHIKV^+^ patients, each genotype was only poorly represented, and a large proportion of them were not observed in healthy donors. In addition, the frequency of genotype #20 was significantly higher in CHIKV^+^ (p = 0.043) and DENV-2^+^ (p = 0.001) patients than in healthy donors (Table 1). Altogether these data suggest that CHIKV and DENV infections were characterized by KIR distributions that differed from healthy controls, suggesting that the KIR repertoire could contribute to an increased susceptibility to CHIKV and/or DENV infection.

**Table 1:** Diversity of the KIR genotypes in CHIKV and DENV2-infected patients compared with healthy controls from the same origin.

Genotype		KIR																CTRL (n = 54)		CHIKV^+^ (n = 30)			DENV-2^+^ (n = 31)		
		2DL1	2DL2	2DL3	2DL4	2DL5	2DS1	2DS2	2DS3	2DS4	2DS5	3DL1	3DL2	3DL3	3DS1	3DP1	3DP2	n	%	n	%	p	n	%	p
1	AA	+	−	+	+	−	−	−	−	+	−	+	+	+	−	+	+	13	24.0	2	6.7		5	16.1	
2	AB/BB	+	−	+	+	+	−	−	−	+	−	+	+	+	−	+	+	3	5.6				1	3.2	
3	AB/BB	+	−	+	+	+	−	−	−	+	+	+	+	+	−	+	+	2	3.7						
4	AB/BB	+	−	+	+	+	+	−	−	+	−	+	+	+	+	+	+	1	1.8						
5	AB/BB	+	+	−	+	+	−	+	−	+	+	+	+	+	−	+	+	3	5.6	2	6.7		1	3.2	
6	AB/BB	+	+	−	+	+	−	+	+	+	−	+	+	+	−	+	+	2	3.7				2	6.4	
7	AB/BB	+	+	−	+	+	+	−	−	+	+	+	+	+	−	+	+	1	1.8						
8	AB/BB	+	+	−	+	+	+	+	−	+	−	+	+	+	−	+	+	1	1.8	1	3.3		1	3.2	
9	AB/BB	+	+	−	+	+	+	+	+	+	−	+	+	+	−	+	+	1	1.8						
10	AB/BB	+	+	−	+	+	+	+	+	+	+	+	+	+	−	+	+	2	3.7				1	3.2	
11	AB/BB	+	+	−	+	+	+	+	+	+	+	+	+	+	+	+	+	1	1.8						
12	AB/BB	+	+	+	+	−	−	+	−	+	−	+	+	+	−	+	+	3	5.6	1	3.3				
13	AB/BB	+	+	+	+	+	−	−	−	+	+	+	+	+	−	+	+	3	5.6	1	3.3		2	6.4	
14	AB/BB	+	+	+	+	+	−	+	−	+	+	+	+	+	−	+	+	3	5.6	2	6.7		2	6.4	
15	AB/BB	+	+	+	+	+	−	+	+	+	−	+	+	+	−	+	+	11	20.3			0.006	1	3.2	
16	AB/BB	+	+	+	+	+	−	+	+	+	−	+	+	+	+	+	+	2	1.8						
17	AB/BB	+	+	+	+	+	+	+	−	+	+	+	+	+	−	+	+	2	3.7	2	6.7				
18	AB/BB	+	+	+	+	+	+	+	+	+	+	+	+	+	+	+	+	1	1.8						
19	AB/BB	+	+	+	+	+	−	+	−	+	+	+	+	+	−	+	+			1	3.3		1	3.2	
20	AB/BB	+	−	+	+	−	−	−	−	+	+	+	+	+	−	+	+			3	10.0	0.043	6	19.3	0.001
21	AB/BB	+	−	+	+	+	+	−	−	+	+	+	+	+	+	+	+			1	3.3		1	3.2	
22	AB/BB	−	+	−	+	+	+	+	−	+	+	+	+	+	−	+	+			1	3.3				
23	AB/BB	−	+	−	+	+	−	+	+	+	+	+	+	+	−	+	+			1	3.3				
24	AB/BB	−	−	−	+	−	−	−	−	+	+	+	+	+	−	+	+			2	6.7				
25	AB/BB	+	+	−	+	+	−	−	−	+	+	+	+	+	+	+	+			1	3.3				
26	AB/BB	+	+	+	+	−	−	−	−	+	−	+	+	+	−	+	+			1	3.3				
27	AB/BB	+	+	−	+	+	−	−	−	+	−	+	+	+	−	+	+			2	6.7				
28	AB/BB	+	+	+	+	+	−	+	−	+	+	+	+	+	+	+	+			2	6.7				
29	AB/BB	+	+	−	+	+	−	−	−	+	+	+	+	+	−	+	+			1	3.3				
30	AB/BB	+	−	+	+	−	−	−	+	+	+	+	+	+	+	+	+			1	3.3				
31	AB/BB	+	−	+	+	−	−	+	−	+	+	+	+	+	−	+	+			1	3.3		1	3.2	
32	AB/BB	+	+	+	+	−	−	+	−	+	+	+	+	+	−	+	+			1	3.3		1	3.2	
33	AB/BB	+	−	+	+	+	+	−	−	+	+	+	+	+	−	+	+						1	3.2	
34	AB/BB	+	+	+	+	+	+	−	−	+	−	−	+	+	−	+	+						1	3.2	
35	AB/BB	+	−	+	+	−	−	+	−	+	−	+	+	+	−	+	+						1	3.2	
36	AB/BB	+	−	+	+	−	−	+	−	+	−	+	+	+	−	+	+						1	3.2	
37	AB/BB	+	−	+	+	−	−	−		+	−	+	+	+	−	+	+						1	3.2	

We next compared individual inhibitory KIR genotypes in healthy volunteers to patients infected with CHIKV or DENV2. Percentages of KIR gene carriers in the Gabonese control samples (Table 2) were in accordance to other African cohorts [22], [23]. In DENV2-infected patients, proportions of all activating and inhibitory KIRs were similar to the healthy control. However, Beltram *et al.* [24] have recently shown for DENV-3 in southern Brazil significant differences for the KIR2DS1, KIR2DS5 and KIR2DL5 genes. The differences with our data could be explained by the DENV serotype (DENV-2 vs DENV-3) and the origin of the populations under study. In contrast, the proportion of KIR2DL1 gene carriers significantly decreased amongst CHIKV^+^ (p^n^ = 0.0338), compared to Gabonese healthy controls. Notably, amongst the “KIR and Diseases Database” (htpp://www.allelefrequencies.net/diseases/) several other diseases were reported to be significantly associated with a modulation of KIR2DL1, including placental Malaria in pregnant Kenyan women [25]. However, for CHIKV-infection, it is important to note that decreased frequency of KIR2DL1 gene is very consistent with the significant cell-surface phenotypic down-modulation of KIR2DL1 inversely associated with the viral load, that we have previously observed in acute CHIKV-infection [5]. Of note, the proportion of KIR2DS5 is significantly increased in CHIKV-infected patients (p^n^ = 0.050) compared to healthy controls (Table 2), as previously observed in patients with HCV infection who cleared the virus in the association with a decreased of KIR2DL2/KIR2DS2 [26]. In addition, the presence of KIR2DS5 appears to be protective in ankylosing spondylitis, endometriosis and acute kidney graft rejection but a lack of KIR2DS5 and presence of C1 allotype was associated with rheumatoid arthritis [27]. KIR2DS5 code for surface receptor that trigger NK cell functions, although its ligand is unknown [28].

**Table 2:** KIR genotypes and HLA ligand combinations in CHIV- and DENV2-infected patients, compared with healthy donors from the same Gabonese population.

	Control		CHIKV^+^				DENV-2^+^			
	n	%	n	%	p	p^n^	n	%	p	p^n^
Inhibitory KIR genotypes										
2DL1	54/54	100	63/73	86.1	0.0049	0.0338	52/52	100	ns	ns
2DL2	35/54	64.8	44/73	60.3	ns	ns	30/52	57.7	ns	ns
2DL3	43/54	79.6	48/73	65.7	ns	ns	41/52	78.8	ns	ns
2DL5	39/54	72.2	40/73	54.8	ns	ns	31/52	59.6	ns	ns
3DL1	54/54	100	73/75	97.3	ns	ns	47/50	94.0	ns	ns
3DL2	54/54	100	68/69	98.5	ns	ns	52/52	100	ns	ns
3DL3	54/54	100	69/69	100	ns	ns	52/52	100	ns	ns
Activating KIR genotypes										
2DL4	54/54	100	68/73	93.1	ns	ns	51/52	98.0	ns	ns
2DS1	10/54	18.5	11/55	20.0	ns	ns	15/52	28.8	ns	ns
2DS2	31/54	64.8	18/39	46.1	ns	ns	16/41	39.0	ns	ns
2DS3	19/54	79.6	7/36	19.4	0.0237	ns	6/36	16.7	ns	ns
2DS4	54/54	100	70/70	100	ns	ns	48/48	100	ns	ns
2DS5	18/54	33.3	33/56	58.9	0.0081	0.0489	23/52	44.2	ns	ns
3DS1	4/54	7.4	7/66	10.6	ns	ns	6/50	12.0	ns	ns
Inhibitory KIR-HLA associations										
C2^+^ in 2DL1^+^	32/47	68.1	44/48	91.6	0.0048	0.0238	26/40	65.0	ns	ns
C1^+^ in 2DL2^+^	20/31	64.5	21/33	63.4	ns	ns	14/22	63.6	ns	ns
C1^+^ in 2DL3^+^	27/38	71.0	18/40	45.0	ns	ns	21/31	67.7	ns	ns
C1^+^ in 2DL2/2DL3^+^	32/47	68.1	28/51	54.9	ns	ns	26/40	65.0	ns	ns
Bw4^+^ in 3DL1^+^	33/47	70.2	38/55	69.1	ns	ns	22/31	59.5	ns	ns
Inhibitory KIR3DL1^+^-HLA-Bw4 subtype associations										
Bw4-80Ile^+^	22/47	46.8	38/55	69.1	0.0274	ns	20/37	54.0	ns	ns
Bw4-80Trn^+^	12/47	25.5	1/55	1.8	0.0005	0.0011	3/37	8.1	0.0472	ns
Activating KIR-HLA associations										
C2^+^ in 2DS1^+^	7/8	87.5	8/8	100	ns	ns	9/12	75.0	ns	ns
C1^+^ in 2DS2^+^	18/22	64.3	12/17	70.6	ns	ns	10/12	83.3	ns	ns

P: Fisher exact test; P^n^: The P values of statistically significant differences were then corrected by the formula P^n^ = 1−(1−P)^n^, where n is the number of comparisons [17].

**Figure 1:**
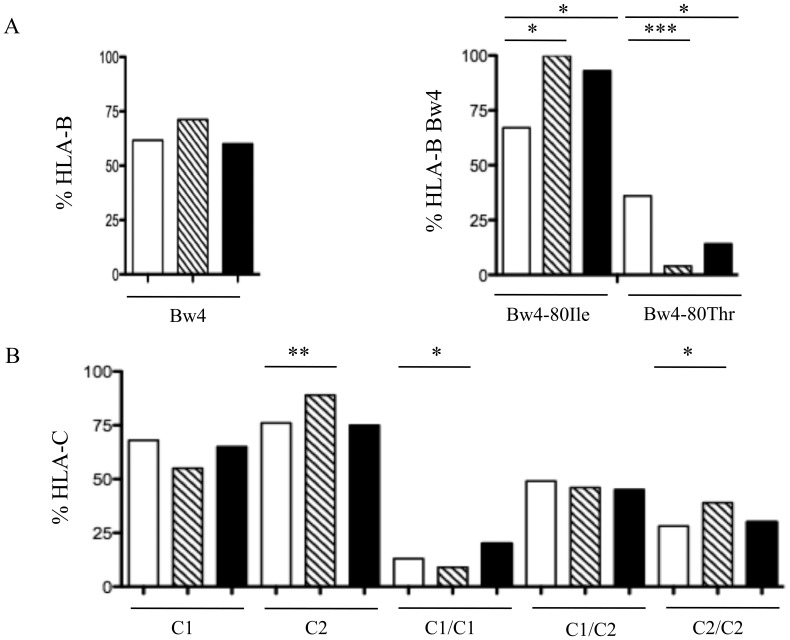
Allele frequency for HLA-B Bw4 (A) and HLA-C (B) subtypes in Gabonese individuals. The frequency of each variable is shown for non-infected controls (n = 54; open bars), CHIKV-infected patients (n = 60; hatched bars), and DENV2-infected patients (n = 45; closed bars). *P<0.05; **P<0.001; ***P<0.0001.

Since the interactions between KIR and their ligands are essential to control of NK cell function, we next evaluated the frequency of KIR genes in combination with the genes encoding their respective known ligands in the same patients. It is noteworthy that all Gabonese individuals, whatever their infectious status, had similar HLA-Bw4 genetic profiles (Figure 1A) which is consistent with what was observed in other African cohorts [29]. However an increased frequency of HLA-B*44, an HLA-Bw4 allele, was recently associated with DENV3 severity in Brazilians [30]. This discrepancy suggests possible differences between the DENV serotypes and/or variations due to ethnicity. HLA-Bw4 (and not Bw6) allotypes serve as ligands for KIR3DL1 subtypes and subtypes are defined by amino-acid variations at positions 77–83 of the HLA-B molecules [7]. Table 2 shows similar proportions of HLA-Bw4 allele amongst KIR3DL1^+^ samples, whatever the study group. HLA-Bw4 molecules are further divided into two groups on the basis of whether isoleucine (Ile) or threonine (Thr) residues are present at position 80, defining HLA-Bw4-80Ile and Bw4-80Thr, respectively [31]. According to Norman et al. [32] an unusual KIR3DL1/S1 evolution occurred in Africans. Thus, KIR3DL1/S1 locus encodes two lineages of polymorphic inhibitory KIR3DL1 allotypes and one lineage of conserved activating KIR3DS1. They also highlighted that random combination of polymorphic KIR3DL1 receptor and HLA-B ligands has vast potential for varying the NK cell response to infection [32]. Figure 1A shows that Bw4-80Thr alleles are significantly more common in CHIKV^+^ patients than in both DENV2^+^ and healthy individuals, individuals. We examined the combination of Bw4-80Ile/Thr and KIR3DL1 in the context of sensitivity to CHIKV or DENV2 infection, and only observed significant modulation for the combination KIR3DL1:Bw4-80Thr (p^n^ = 0.001), compared to healthy controls (Table 2). In agreement with receptor-binding and lysis-inhibition data suggesting that HLA-Bw4-80Ile molecules are more effective ligands for KIR3DL1 than HLA-Bw4-80Thr [33], we have observed no significant phenotypic modulation of KIR3DL1 expression after acute CHIKV [5] and DENV2 (Petitdemange *et al.*, Manuscript in preparation) infections. In contrast, for HIV-1 the KIR3DL1:HLA-Bw4-80Ile combination was described in association with disease severity [34].

HLA-C molecules are also dichotomized into two groups, based on their KIR specificity; HLA-C group 1 (C1) specifically binds to KIR2DL2/DL3 and HLA-C group 2 (C2) binds to KIR2DL1 [8]. The genetic combinations of KIR2DL2/DL3 or KIR2DS2 and HLA-C1 genes remained similar in infected and healthy Gabonese yet we have previously reported a clonal expansion of NK cells that co-express activating CD94/NKG2C and inhibitory KIR2DL2/DL3 receptors in direct association with the viral load during acute CHIKV-infection [5]. The combination of KIR2DL2/DL3 with HLA-C1 might therefore not be involved in susceptibility to CHIKV infection but the observed expansion of NK cells expressing these receptors suggests that this interaction is of importance during the development of the disease. For example, KIR2DL2/DL3:HLA-C1 interaction could be associated with the delayed progression to the development of persistent chronic inflammation in CHIKV-infected patients. Infiltrating activated NK cells were found in synovial tissue in close vicinity to chronically CHIKV-infected perivascular synovial macrophages [35]. Importantly, a significant increase in frequency of HLA-C2 was observed in CHIKV-infected patients (p = 0.0041), mainly of HLA-C2 homozygous subjects, compared to DENV^+^ patients and controls (Figure 1B). More intriguingly, this HLA-C2 increased effect was also significantly observed in combination with KIR2DL1 (p = 0.0238) (Table 2). In this regard, however, it cannot be ruled out that these results were influenced by population demographic history; these results will need to be confirmed through the study of samples collected from other ethnic groups. Notably, this data highlights that CHIKV infection could be influenced by KIR2DL1/HLA-C2 interplay. Consistent with this notion we have previously shown a depletion of KIR2DL1^+^ cells early after CHIKV infection, inversely associated with the viral load [5].

The finding that inhibitory interactions are protective against a viral infection initially seems counterintuitive. However, NK cells are held in check by their inhibitory receptors as originally proposed by the missing-self hypothesis^,^ and loss of these interactions could be a key mechanism to allow NK cell activation [36]. *In vitro* studies with autologous influenza infected targets have shown that NK cells from individuals with KIR2DL3:HLA-C1 were activated more rapidly than those with KIR2DL1:HLA-C2 [37]. Consistently we previously showed that the KIR2DL1^+^ NK cell depletion was associated with an expansion of KIR2DL2/DL3^+^ NKG2C^+^ cells after acute CHIKV infection [5]. In summary, we can hypothesize that the expansion of highly functional NK cells and the development of a strong adaptive memory response, as previously described [5], [38], are independent of a specific KIR/HLA pathway in DENV2 infection, but certainly associated to an interplay between KIR2DL1 and HLA-C2 in response to CHIKV infection. This study is an interesting first step towards understanding the different roles of KIR and their specific ligands in CHIKV and DENV2 infections. However, further studies will be necessary to conclude as to the role of these receptors and ligands in the context of susceptibility to these infections or in the development of the disease and chronic or hemorrhagic symptoms.
